# Restoration of the Inner Blood–Retinal Barrier in Diabetic Retinopathy Through Norrin-FZD4 Signaling

**DOI:** 10.3390/ijms27219849

**Published:** 2026-11-03

**Authors:** Laura González-González, David A. Antonetti

**Affiliations:** Department of Ophthalmology and Visual Sciences, Kellogg Eye Center, University of Michigan Ann Arbor, MI 48105, USA; gonzalla@med.umich.edu

**Keywords:** blood–retinal barrier, diabetes, retinopathy, norrin, Wnt/β-catenin

## Abstract

Diabetic retinopathy (DR) is a leading cause of irreversible vision loss in the working-age population and is characterized by the disruption of the blood–retinal barrier (BRB) integrity and increased vascular permeability. Elevated vascular endothelial growth factor (VEGF) contributes to BRB dysfunction in DR. Current therapies for DR, including anti-VEGF agents, have provided significant clinical benefits; however, not all patients respond optimally, highlighting the need for new therapeutic approaches. Norrin, an endogenous cytokine essential for blood–retinal barrier development and maintenance, has emerged as a potential therapeutic target for restoring barrier function in retinal vascular disease. In this review, we summarize the evidence supporting norrin signaling as a mechanism for restoring BRB properties following VEGF-induced barrier disruption and in DR, as well as its role in preserving visual function. We also discuss the development of engineered FZD4 agonists designed to mimic or enhance norrin signaling, providing a therapeutic strategy to activate this pathway independently of endogenous norrin. Together, these findings highlight norrin-FZD4 signaling as a potential therapeutic approach to restore BRB integrity and preserve vision in DR and other blinding diseases associated with BRB dysfunction.

## 1. Introduction

The blood–retinal barrier (BRB) is a tightly regulated, highly selective barrier that maintains the specialized environment of the neural retina necessary for vision. The BRB regulates the exchange of molecules between the circulation and the retinal parenchyma, thereby protecting the neural tissue from harmful blood-borne substances and supplying the retina with oxygen and essential nutrients. Structurally, the BRB is subdivided into outer (oBRB) and inner (iBRB). The oBRB is located at the outermost part of the retina under the photoreceptor layer and comprises the retinal pigment epithelium (RPE) that separates Bruch’s membrane and the underlying choriocapillaris from the retina and its function is to ensure nutrient transport and filtration of metabolic waste products to maintain photoreceptor function while maintaining the photoreceptor environment. The iBRB is formed by specialized retinal endothelial cells surrounded by a double basement membrane and closely associated with neurons, pericytes, astrocytes, Müller cells, and microglia that collectively regulate vascular permeability and are referred to as the neurovascular unit (NVU) [1]. The barrier function depends on the coordinated regulation of paracellular and transcellular transport across the iRBR and oBRB. The paracellular pathway is restricted by endothelial tight junction (TJ) and adherence junctional (AJ) complexes between cells and low transcellular permeability is maintained by limited vesicular transport while transporter all regulated flux of nutrients [2]. Disruption of BRB integrity is a hallmark of several debilitating, blinding retinal diseases, including diabetic retinopathy (DR), and current therapies preventing BRB loss are effective in maintaining vision.

DR is a common vision-threatening complication of both type 1 and type 2 diabetes and a leading cause of irreversible blindness in the working-age population [3] and is clearly associated with metabolic dysregulation and hyperglycemia [4]. Non-proliferative diabetic retinopathy (NPDR) is characterized by increased iBRB permeability, pericyte loss, lipid exudates, hemorrhages and edema with cystoid macular edema most closely linked to vision loss [5]. Proliferative diabetic retinopathy (PDR) involves persistent vascular dysfunction, retinal hypoxia and local ischemia promoting pathological neovascularization into the vitreous, vitreous hemorrhaging, retinal detachment and blindness [5]. In DR, retinal vascular permeability and pathological angiogenesis are driven in part by elevated levels of vascular endothelial growth factor (VEGF) and therapies targeting VEGF relieve diabetic macular edema (DME) and inhibit neovascularization and are effective in preventing vision loss [6]. While advances in binding VEGF or binding related vasoactive factors such as angiopoietin 2 (Ang-2) along with VEGF have provided important advances, no new targets for treating DR have evolved since the development of anti-VEGF therapies. After a brief review of VEGF signaling as background, we will review the literature demonstrating norrin as a necessary cytokine for BRB formation or barriergenesis and maintenance. We will then detail studies of the norrin signaling pathway as a potential target both to prevent BRB loss and restore barrier properties following VEGF-induced disruption or in DR, as well as discuss ongoing clinical trials.

## 2. VEGF and BRB Disruption

VEGF is a secreted glycoprotein and a key regulator of angiogenesis, with essential roles in vascular development [7] and has been identified as an important driver of and pathological processes in retinal eye disease, including retinal vein occlusions, retinopathy of prematurity and DR. In DR, increased VEGF levels in the serum and aqueous humor correlate with disease severity, with higher concentrations in patients with PDR than in non-PDR patients [8].

VEGF disrupts the organization of endothelial TJ proteins, including occludin (OCLN), claudin-5 (CLDN5), zonula occludens-1 (ZO-1), and AJ proteins such as VE-cadherin, compromising BRB integrity [9]. A key mechanism involves VEGF-induced activation of protein kinase Cβ (PKCβ), which promotes phosphorylation of occludin at Ser490. This phosphorylation drives two internalization pathways. A recent study reveals that OCLN binds to the motor protein dynein acting as an adaptor for TJ trafficking as a vesicle and disassembly of the TJ [10]. Also, Ser490 phosphorylation of OCLN can increase ubiquitination and cytoplasmic translocation of OCLN, thereby leading to disassembly of the junctional complex and increased vascular permeability [11,12]. Beyond its effect on intercellular junctions, VEGF also increases transcellular transport by promoting the expression of caveolin-1 and plasmalemma vesicle-associated protein (PLVAP), further contributing to BRB breakdown [1,9]. In summary, VEGF drives both paracellular permeability by regulating TJ and AJ trafficking away from the cell border and junctional complex disassembly and promotes transcellular flux, collectively promoting loss of the BRB and contributing to macular edema. In addition, VEGF acts as a critical driver of both developmental angiogenesis and neovascularization.

## 3. DR Therapeutic Management

The central role of VEGF in BRB disruption has established anti-VEGF agents as the first-line treatment for DR, with ranibizumab, bevacizumab, and aflibercept currently used and recently faricimab, which targets both VEGF and Ang-2, has been approved for therapy. Although anti-VEGF therapy has demonstrated significant clinical efficacy in preventing vision loss, it is limited to patients with overt vascular pathology, requires several intravitreal (IVT) injections and maintenance therapy [13], and about 50% of patients fail to respond [14], while others lose the initial acuity gains due to developing resistance [15]. Importantly, anti-VEGF therapy prevents further vascular damage, slowing disease progression rather than restoring pathological blood vessels.

Additional therapeutic approaches for DR include laser photocoagulation and corticosteroid administration. Although focal or grid laser photocoagulation can reduce the risk of moderate vision loss, it rarely improves vision and may cause collateral retinal damage. Corticosteroids such as IVT triamcinolone, dexamethasone, and fluocinolone acetonide implant act through the activation of the glucocorticoid receptor to reduce pro-inflammatory cytokines and chemokines, leukostasis, microglial activation, and Müller cell-mediated inflammation [16]. They also downregulate VEGF production and promote the re-establishment of TJ proteins, including OCLN and CLDNs, thereby supporting BRB integrity. However, long-term use is associated with cataract progression and elevated intraocular pressure, limiting corticosteroids to second-line therapy in DR [16].

Given the limited response and adverse effects to current DR treatments, and since it is estimated that the number of individuals affected by DR will increase by 55.6%, from 103.12 million in 2020 to 160.50 million in 2045 [17], the development of new therapeutic approaches that target earlier stages of the disease and preserve visual function is urgently needed.

Restoring the iBRB is emerging as a promising therapeutic approach for early-stage DR. Supporting this concept, conditional expression of a VEGF non-phosphorylatable occludin mutant (S490A) specifically in vascular endothelial cells prevents diabetes-induced BRB permeability and leukostasis in mice, and importantly, completely preserves visual acuity and contrast sensitivity compared to diabetic control animals [18]. These findings underscore the critical role of BRB integrity in DR and suggest that therapies focused on restoring barrier function may not only limit vascular leakage but also preserve visual function. The mechanisms that establish and maintain the retinal BRB may provide insights into new therapeutic strategies for treating DR.

## 4. Norrin Signaling in Retinal Development

Canonical Wnt signaling is critical for both developmental angiogenesis in the retina and central nervous system and for the establishment and maintenance of the blood–brain barrier (BBB) and BRB [19,20]. While Wnt7a and Wnt7b are the primary canonical Wnt ligands involved in BBB development, norrin is the predominant ligand activating canonical Wnt signaling in the retina and cerebellum [21].

Norrin is a secreted protein of 131 amino acids. Although Müller cells are considered the principal source of norrin in the retina [22,23], *Ndp* mRNA has also been detected by in situ hybridization in the retinal ganglion cell (RGC) layer and inner nuclear layer [24]. Single-cell transcriptomic analysis further revealed high *Ndp* expression in horizontal cells [25] and subsets of amacrine cells [26].

Norrin binds to the *N*-terminal extracellular cysteine-rich domain of Frizzled 4 (FZD4) and the β-propeller domains of the low-density lipoprotein receptor-related protein 5/6 (LRP5/6) and the large extracellular loop of the tetraspanin (TSPAN12) co-receptor, activating canonical Wnt/β-catenin signaling [27]. TSPAN12 selectively enhances β-catenin signaling induced by norrin but not by other Wnt ligands [28]. In the absence of norrin or other Wnt ligands, the APC destruction complex, composed of adenomatosis polyposis coli (APC), axin, casein kinase 1 α (CK1α), protein phosphatase 2A (PP2A) and glycogen synthase kinase 3α/β (GSK-3α/β), promotes β-catenin phosphorylation for its subsequent ubiquitination and proteasomal degradation, maintaining low levels of cytoplasmic β-catenin. After FZD4 receptor complex activation, disheveled (DVL) recruits the APC complex to the plasma membrane, inhibiting the activity of the destruction complex. This prevents β-catenin degradation, allowing its cytoplasmic accumulation and nuclear translocation. In the nucleus, β-catenin associates with the T-cell factor/lymphoid enhancer-binding factor (TCF/LEF) complex to promote the transcription of Wnt target genes [29]. This transcriptional program is essential for proper vascular development. In mice, deletion of β-catenin results in embryonic lethality due to fragile and malformed vessels with abnormal lumen and hemorrhages [30]. Inhibition of β-catenin signaling in endothelial cells at postnatal stages results in reduced CLDN3 and 5, which are required to restrict paracellular permeability, and increased PLVAP, thereby compromising BBB properties [31].

The critical role of norrin signaling in retinal angiogenesis and BRB establishment emerged from studies of Norrie disease and familial exudative vitreoretinopathy (FEVR). Norrie disease is an X-linked disorder caused by mutations in the *NDP* gene, which encodes norrin and leads to congenital or early childhood blindness [32,33]. In humans, mutations in the genes that code for FZD4, LRP5, TSPAN12, β-catenin, and norrin cause FEVR, a disorder characterized by incomplete and aberrant vascularization of the peripheral retina, neovascularization, retinal folds and detachment, and vitreous hemorrhage resulting in total blindness [34]. In mice, the silencing of the norrin, FZD4, LRP5, or TSPAN12 genes phenocopies the retinal hypovascularization observed in Norrie disease and FEVR [20,22,28]. In addition, these retinas exhibit high retinal vascular permeability, which correlates with reduced CLDN5 border staining and increased PLVAP expression [20,35]. Importantly, expression of a stabilized form of β-catenin largely reverses the vascular defects [20]. However, overexpression of the stabilized β-catenin in regions with low barrier properties, such as the circumventricular organ in the brain and choriocapillaris of the eye, fails to completely induce the BBB and BRB [36], suggesting that additional pathways are important. These findings demonstrate that norrin signaling is essential not only for retinal angiogenesis but also for the establishment of the BRB.

Norrin knockout (KO) mice exhibit a complete absence of intraretinal capillaries [23], and these vascular abnormalities result in blindness. However, ectopic transgenic expression of norrin from the lens [37] or AAV-9-mediated *NDP* delivery [38] restores normal retinal vascular development. Additionally, AAV vectors targeting Müller glia or RGCs restore intraretinal capillary growth, preserve BRB integrity, and rescue visual function [39]. In the mouse model of oxygen-induced retinopathy (OIR), ectopic norrin expression or administration of recombinant human norrin promotes vascular regrowth and prevents the formation of pathological neovascular tufts [40,41]. Norrin-induced vascular regrowth has been linked to increased Ang2 expression, which along with VEGF, promotes endothelial cell proliferation [40] and, together with its role in maintaining BRB integrity, may contribute to the restoration of a functional retinal vasculature, reducing retinal hypoxia and the associated stimulus for pathological growth. The ability of norrin to promote the restoration of the retinal vasculature network after injury is particularly relevant to retinal diseases characterized by abnormal vessel growth, neovascularization, and severe visual impairment, including retinopathy of prematurity, age-related macular degeneration, and DR.

## 5. Norrin Signaling in BRB Restoration

Norrin expression is not restricted to retinal development and persists in the adult retina. In the adult mouse, disruption of norrin/FZD4/β-catenin signaling compromises BRB integrity, highlighting its contribution to barrier properties maintenance [35]. FZD4 signaling activation promotes the expression of key regulators of the specialized BRB and BBB phenotype such as major facilitator superfamily domain containing 2 (MFSD2), CLDN5 and ZO-1, while suppressing PLVAP expression [21,36,42,43], indicating that FZD4 signaling inhibits vascular leakage. These changes promote the formation of the endothelial TJs and limit the transcellular permeability, two essential characteristics of the BRB.

**Figure 1:**
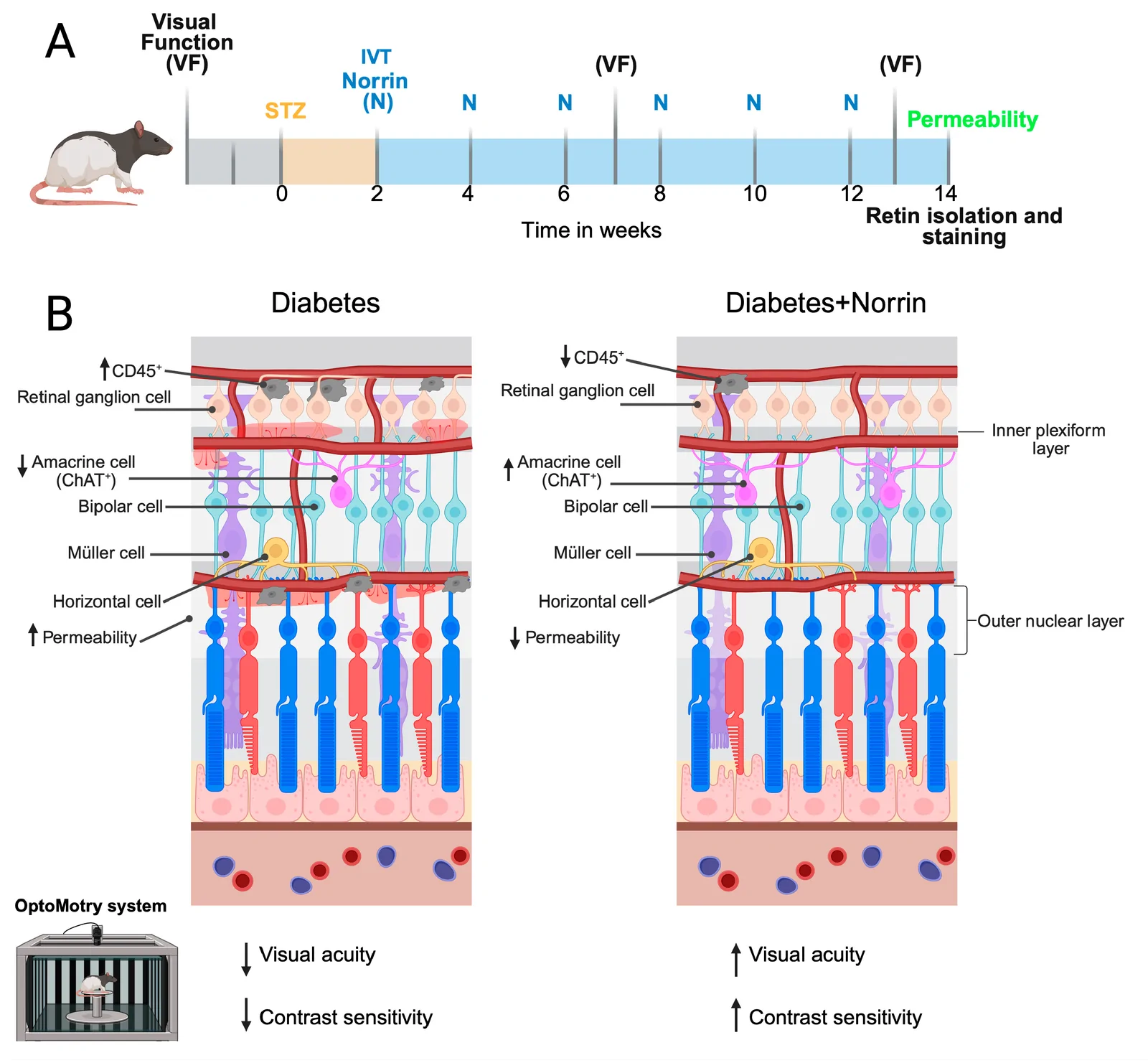
Norrin prevents vascular permeability and amacrine cell loss and preserves visual function in diabetic rats. (**A**) Schematic of the norrin intervention trial. Long–Evans rats received six intravitreal (IVT) injections of norrin (40ng/eye) beginning 2 weeks after STZ-diabetes induction at 2-week intervals. Visual function was assessed using the OptoMotry system, vascular permeability by intravenous injection of FITC-BSA, and retinal cell loss by immunostaining for specific retinal cell type markers, including choline acetyltransferase (ChAT) for cholinergic amacrine cells and CD45 for inflammatory cells. (**B**) Norrin intervention prevents vision loss in STZ-induced diabetic rats. Norrin treatment reduced albumin leakage, decreased the number of retinal CD45+ inflammatory cells, preserved the number of amacrine ChAT+ and preserved visual acuity and contrast sensitivity in diabetic rats. Upward and downward arrows indicate increases and decreases, respectively, in the corresponding parameters relative to non-diabetic control animals. Created in BioRender. Gonzalez Gonzalez, L. (2026) https://BioRender.com/w8si4z2 (accessed on 16 October 2026).

Consistent with its role in maintaining BRB properties, IVT administration of norrin reduces retinal vascular leakage in models of VEGF-induced permeability and STZ-induced diabetic rats and mice [44,45]. In rats 5 months post-diabetes induction, retinal norrin protein levels are reduced, coinciding with decreased visual acuity and contrast sensitivity [46]. Norrin IVT administration (40 ng/eye) starting 2 weeks post-STZ injection prevented diabetes-induced visual dysfunction while blocking the diabetes-induced increased vascular permeability, CD45+ inflammatory cell accumulation, and neural cell death [46]. Retinal neural cell apoptosis is observed in animal models of DR and in humans [47], and specifically, loss of retinal choline acetyltransferase-positive (ChAT^+^) amacrine cells has been observed in models of DR. Norrin treatment prevented this loss of ChAT^+^ amacrine cells in STZ-induced diabetic rats [46] (Figure 1). In contrast, high-dose norrin treatment (200 ng/eye) beginning 8 weeks after diabetes induction, when BRB dysfunction is already established, reduces vascular permeability and preserves visual function [46]. These findings suggest that norrin can both prevent the development of BRB dysfunction when administered early and promote the restoration of barrier properties when administered after dysfunction has been established.

Additional evidence from an STZ-induced diabetic mouse model indicates that norrin IVT administration reduces VEGF overexpression and restores pigment epithelium-derived factor (PEDF) levels, and protected retinal ganglion cell (RGCs) from apoptosis [45]. Beyond diabetic models, norrin signaling increases the number of surviving RGC perikarya and optic nerve axons and, together with endothelin-2, protects photoreceptors from light-induced damage [48,49]. However, despite these findings, single-cell transcriptomic analysis indicates that *Fzd4* expression in the retina is largely restricted to vascular endothelial cells [25,50]. This suggests that in diabetic animal models, norrin preserves neural cells downstream of BRB restoration rather than through direct signaling in neural cells.

## 6. Molecular Mechanism of Norrin-Mediated BRB Restoration

Norrin promotes barrier properties through both a canonical and non-canonical pathway. Previous studies demonstrated that norrin restores barrier properties in bovine retinal endothelial cells (BREC) coincident with or after VEGF stimulation without altering VEGF signaling. Instead, VEGF promotes norrin signaling by inducing the expression and membrane localization of the co-receptor TSPAN-12 through a MEK/ERK-dependent mechanism [44]. These studies also revealed the requirement for the canonical pathway as inhibition of this pathway with XAV-939, which stabilizes axin and promotes β-catenin degradation, reduced the effect of norrin on barrier restoration consistent with in vivo studies revealing that vascular endothelial cell expression of constitutively active β-catenin could largely restore BRB properties [20]. However, activation of β-catenin signaling with BIO, a GSK-3 α/β inhibitor, alone is not sufficient to restore the barrier after VEGF in BREC even though β-catenin activation was clearly demonstrated [44]. These findings demonstrated that norrin-induced barrier restoration involves additional mechanisms beyond canonical β-catenin signaling.

In addition to canonical β-catenin signaling, norrin promotes endothelial barrier restoration through a novel, non-canonical pathway involving the direct interaction between DVL1 and CLDN5 [51]. Mammals express three DVL proteins, encoded by three distinct genes (DVL1, 2, and 3), with a general structure that contains a DIX (DVL and axin), a PDZ domain (post-synaptic density protein-95, disc large tumor suppressor, zonula occludens-1), and a DEP domain (DVL, EGL-10, and pleckstrin) and at the C-terminus, a PDZ-binding motif (PDZ-BM) [29]. Previous studies demonstrate that deletion of *Dvl2* or *Dvl3* results in cardiac outflow tract abnormalities with partial functional redundancy among DVL proteins. DVL1 is widely expressed in the brain, and studies in *Dvl1* KO mice reported abnormalities in social behavior and sensorimotor gating, while human DVL1 mutations have been associated with neurocognitive and neurodevelopmental disabilities, suggesting an important role in central nervous system function and highlighting the distinct functions of individual DVL proteins [52,53]. In adult rat retinal capillaries and BREC, DVL1 and 3 are expressed and colocalize with CLDN5 and ZO-1 at the cell border and both immunoprecipitation and expression of tagged protein/pull down assays revealed that DVL1 interacts with CLDN5 [51]. Silencing of *Dvl1* but not *Dvl2* or *Dvl3* reduces BREC barrier properties and prevents norrin barrier restoration after VEGF treatment with reduced CLDN5 localization at endothelial cell borders. These findings indicate a specific role for DVL1 in barrier maintenance and restoration that cannot be compensated by DVL3 [51].

**Figure 2:**
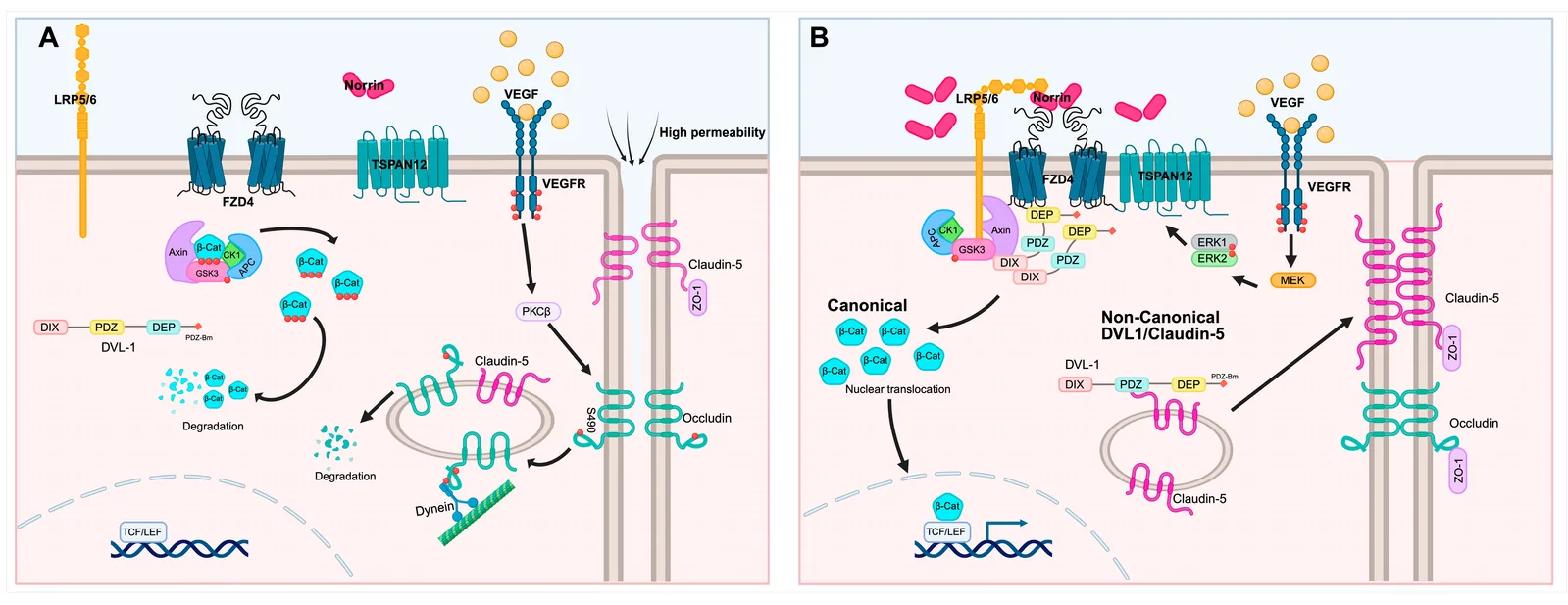
Norrin-induced endothelial barrier restoration scheme. (**A**) VEGF induces serine 490 phosphorylation (S490) of occludin by PKCβ, leading to occludin internalization and binding to dynein to allow trafficking of tight junction proteins as vesicular cargo and increased permeability. (**B**) VEGF induces endothelial permeability and simultaneously promotes norrin signaling by inducing FZD4 co-receptor TSPAN12 migration to the plasma membrane. Norrin signals through DVL1, promoting the β-catenin canonical pathway and a non-canonical pathway involving the interaction between DVL1 and CLDN5 to promote barrier restoration. Black arrows indicate the direction of signaling events, protein trafficking, or molecular interactions. Red dots indicate phosphorylation sites. Created in BioRender. Gonzalez Gonzalez, L. (2026) https://BioRender.com/0uqrqni (accessed on 16 October 2026).

Solution nuclear magnetic resonance spectroscopy and competition-binding studies have revealed that the C-terminal PDZ-BM of DVL1 binds with high affinity to its PDZ domain, forming a closed-loop structure [29], likely occurring on DVL2 and 3 as well. Pull down assays reveal that DVL1 interacts with CLDN5 through its PDZ domain, suggesting that CLDN5 and the DVL1-CT compete for binding to this region and this was confirmed in pull down assays as well. Accordingly, overexpression of the DVL1-CT peptide on BREC disrupts the DVL1-CLDN5 interaction, decreases CLDN5 staining at the cell borders and prevents Norrin-induced barrier restoration following VEGF treatment [51]. Collectively, these data support a non-canonical pathway for DVL1 and CLDN5 interaction in regulating the retinal endothelial barrier. However, the physiological relevance of this alternative pathway remains to be established in vivo. In summary, norrin signaling through the canonical pathway promotes β-catenin driven transcription required for barrier properties but also promotes DVL1 and CLDN5 interaction necessary for proper assembly of the junctional complex that can be blocked either by *Dvl1* KO or by competing away DVL1-CLDN5 interaction by overexpressing the DVL1 PDZ-BM (Figure 2).

Although canonical β-catenin signaling is a well-established component of norrin signaling, current evidence indicates that its activation alone is not sufficient to restore BRB properties following vascular injury. This highlights the importance of fully identifying the mechanisms involved in norrin-induced barrier restoration. In particular, the interaction between DVL1 and CLDN5 represents a novel mechanism through which norrin may directly regulate tight junction organization. Further characterization of this and other possible non-canonical pathways may provide a more complete understanding of how norrin restores endothelial barrier properties and identify additional therapeutic targets for BRB restoration.

## 7. Engineered FZD4 Agonists as Alternatives to Norrin for BRB Restoration in DR

Although activation of the norrin signaling pathway has significant potential for restoring BRB integrity and preserving visual function in DR, the use of exogenous, recombinant norrin is limited by its poor secretion and its strong association with the extracellular matrix [54]. Moreover, norrin is a small, disulfide-rich protein containing 11 cysteines with disulfide bonds that are essential for the tertiary structure of the monomer and the stability of the active dimer [27]. These structural characteristics make norrin difficult to produce and purify at high concentration from mammalian or insect cells. Expression of Norrin in *E.coli* results in the formation of inclusion bodies, requiring protein refolding to recover biological activity [55]. These limitations have driven the development of alternative strategies to selectively activate the FZD4/β-catenin signaling, including engineered FZD4 agonists designed to mimic norrin activity. These agonists are described below, and their molecular design, mechanism of action, and clinical development stages are summarized in Table 1.

### 7.1. P6C.51.61

Norrin activates Wnt/β-catenin signaling by engaging FZD4 and the E1E2 domains of the LRP5/6 co-receptor [27]. P6C.51.61 is an anti-LRP5 antibody that binds the E3E4 domains of LRP5, a region distinct from the norrin binding site, and promotes formation of the norrin/FZD4/LRP5 complex, thereby enhancing downstream signaling. In vivo, P6C.51.61 partially rescues retinal vascular defects in *TSPAN12*-deficient mice and reduces pathological neovascularization in the OIR model [54]. Since other Wnt ligands are present in the vitreous humor, it remains unclear whether the effects of P6C.51.61 are specific to enhanced norrin signaling. Moreover, because P6C.51.61 enhances receptor complex formation rather than directly providing a ligand, its efficacy may depend on the availability of endogenous ligands. This may represent a limitation for its potential use in DR, where endogenous norrin levels are reduced by diabetes [46].

### 7.2. SZN-413 and SZN-8141

SZN-413 is a tetravalent bispecific molecule consisting of an antibody that specifically binds to the extracellular cysteine-rich domain of FZD4, and an LRP5-biased binder fused to the antibody light chain. By bringing FZD4 and LRP into proximity, SZN-413 promotes Wnt/β-catenin signaling in endothelial cells as evidenced by the increased expression of the target genes *LEF1, AXIN2*, *MFSD2A* and induces a higher expression of ZO-1 and CLDN5, demonstrating that this tetravalent antibody can increase vascular integrity through upregulation of barrier function proteins. SZN-413 inhibits pathological neovascularization and promotes proper retinal vessel regrowth in an OIR mouse model and prevents the retinal leakage induced by VEGF in a rabbit model [50]. SZN-8141 is a fusion molecule that combines a norrin mimetic with an anti-VEGF molecule that reduces both neovascular and avascular areas in OIR model [56]. SZN-8141 has recently advanced to DUET a phase 1b/2a clinical trial in patients with DME to evaluate its safety and tolerability [57].

### 7.3. F4L5.13

F4L5.13 is a tetravalent antibody that contains two diabodies, each having two binding sites for FZD4 and LRP5. F4L5.13 was designed to simultaneously engage FZD4 and LRP5 for the activation of the Wnt/β-catenin signaling in a TSPAN12-independent manner [42]. In mouse brain endothelial cells, F4L5.13 induces the expression of ZO-1, CLDN3, and CLDN5 after VEGF-induced barrier disruption [42]. In *Tspan12*-KO mice with dramatic loss of deep capillary formation and BRB properties, F4L5.13 i.p. injection restores retinal angiogenesis and promotes barrier function by increasing *Cldn5* and reducing *Plvap* expression. In the OIR model, F4L5.13 reduces pathological neovascular tuft formation, although it does not reduce the avascular area [42]. The IVT administration of F4L5.13 also restores BRB function in adult mice with inducible endothelial cell-specific Tspan12-KO [58]. The translational relevance of this approach is highlighted by the development of MK-3000 (Restoret), an optimized version of F4L5.13. MK-3000 has been evaluated in the phase 1b/2a AMARONE clinical trial, where it showed a safety profile and preliminary improvements in visual and anatomical outcomes in patients with DME [59] (for further details, see [60]). MK-3000 has advanced to phase 2b/3 clinical trials BRUNELLO and BAROLO, which compare the efficacy of Restoret with ranibizumab in patients with DME [61,62]. The SUPER TUSCAN trial will assess visual acuity and retinal morphology in patients with neovascular age-related macular degeneration (AMD) or macular edema following branch retinal vein occlusion receiving MK-3000 as monotherapy or in combination with aflibercept, an anti-VEGF agent [63].

**Table 1:** Engineered FZD4 agonists in preclinical and clinical development. The table summarizes the molecular design, mechanism of action, and development stage. Schematic representations were adapted from [54] and Created in BioRender. Gonzalez Gonzalez, L (2026) https://BioRender.com/3ymntbt (accessed on 16 October 2026).

Engineered FZD4 Agonist	Molecular Design	Mechanism of Action	Development Stage	Reference
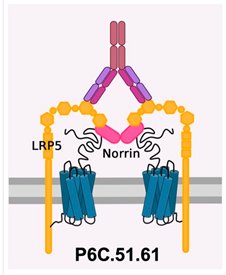	Anti-LRP5 E3E4 antibody	Promotes assembly of the norrin/FZD4/LRP5 complex	Preclinical	[54]
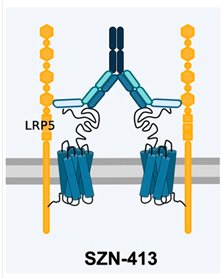	Tetravalent bispecific that binds to FZD4 cysteine-rich domain and LRP5	Promotes assembly of the FZD4/LRP5 complex activating the receptor	Preclinical	[50]
**SZN-8141**	Combines a norrin mimetic with an anti-VEGF molecule	Bi-specific approach norrin mimetic and anti-VEGF	Phase 1b/2a DUET (NCT07817121)	[56,57]
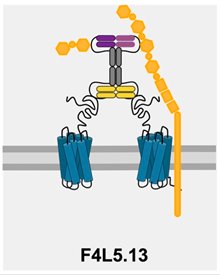	Tetravalent antibody with two diabodies, each with two binding sites for FZD4 and LRP5	Brings FZD4 and LRP5 into proximity activating the receptor	Optimized version MK-3000 (Restoret) Phase 1b/2a AMARONE (NCT05919693) Phase 2b/3 BRUNELLO (NCT06571045) BAROLO (NCT06957080) SUPER TUSCAN (NCT07205887)	[58,59,61,62,63]

## 8. Additional Considerations on β-Catenin Signaling in DR

Although extensive evidence supports that the β-catenin signaling pathway is essential for retinal vascular development and for BRB restoration in diabetes, previous studies have also implicated Wnt/β-catenin signaling as a pathogenic pathway in DR. Previous reports showed increased β-catenin levels and nuclear localization in post-mortem retinal sections from patients with DR, as well as high LRP5/6 receptor levels in murine models of diabetes and OIR. Notably, the increased β-catenin and LRP5/6 were predominantly observed in the inner retina. Furthermore, hyperglycemic conditions induced β-catenin nuclear accumulation in cultured retinal endothelial cells. In vivo IVT injection of DKK1, a Wnt pathway inhibitor, reduced retinal vascular leakage in STZ-diabetic rats [64]. Similarly, blocking LRP6 with the monoclonal antibody Mab2F1 attenuated β-catenin accumulation and reduced retinal permeability [65]. Altogether, this evidence has suggested that excessive Wnt/β-catenin contributes to the pathogenesis of DR and shows a discrepancy with the data in this review. This discrepancy may reflect differences in ligand-receptor engagement, cellular context, subcellular localization, and duration of β-catenin pathway activation. For example, single-cell sequencing studies have shown that LRP6 is widely expressed across multiple retinal cell types [25], suggesting that LRP6 blockade may affect Wnt signaling in both vascular and non-vascular cells. Although other Wnt molecules such as Wnt3a, Wnt7a, and Wnt10a have been detected in the vitreous [66], whether these ligands contribute to the broader activation of β-catenin signaling observed in DR remains unknown. Supporting the importance of cellular context, overexpression of the stabilized β-catenin in mature endothelial cells did not alter CLDN5 or PLVAP expression in the retinal vasculature, although it modified barrier-associated gene expression in choriocapillaries [36]. Thus, selective activation of β-catenin signaling through the norrin/FZD4 receptor complex may provide a targeted approach to promote BRB integrity, given the largely restricted expression of *Fzd4* in retinal endothelial cells. The specific activation may minimize the potential deleterious effects associated with broader activation of the pathway. However, the long-term consequences of sustained β-catenin activation in the retina remain to be characterized.

## 9. Conclusions and Future Directions

Disruption of the BRB is a central feature of DR and contributes to retinal inflammation, neuronal dysfunction, and progressive vision loss. Although current therapies, such as VEGF inhibitors, have substantially improved the treatment of diabetic retinal disease, they primarily target ongoing vascular pathology and do not directly restore the BRB. Therefore, strategies that promote the restoration and maintenance of BRB integrity may provide an important therapeutic approach for earlier stages of DR, with the potential to preserve retinal function and protect vision.

Norrin signaling plays a critical role in the establishment and maintenance of the retinal vasculature and BRB. Importantly, accumulating evidence indicates that activation of this pathway can also restore barrier properties after barrier integrity injury in in vivo and in vitro models. In the diabetic rat model, the reduction in vascular permeability by norrin is accompanied preservation of visual function as well prevention of neural cell death and increased inflammatory cells. These findings suggest that restoring the BRB as an important therapeutic target to prevent vision loss in DR.

Despite norrin therapeutic potential, the direct use of recombinant norrin is limited by the difficulty of producing the biologically active protein. These limitations have driven the development of alternative strategies to activate the norrin signaling pathway, including engineered FZD4 agonists that mimic the angiogenic and barrier-restoring effects of norrin in developmental and ischemic models, such as OIR. However, whether engineered FZD4 agonists can reproduce the barrier effects of norrin in diabetes remains to be determined and is currently under investigation. Indeed, recently released data from the Brunello phase 2b/3 trial reveal that MK-3000 (Restoret) was not inferior to ranibizumab and was effective in treatment of DME at 1 year (BRUNELLO Trial (NCT06571045)). These are progressing to clinical trials and hold promise as a novel therapeutic approach to treat DR and other vision-threatening diseases involving retinal vascular permeability.

## Data Availability

No new data were created or analyzed in this study. Data sharing is not applicable to this article.

## Abbreviations

The following abbreviations are used in this manuscript:

BRB	Blood–retinal barrier
oBRB	Outer BRB
iBRB	Inner BRB
RPE	Retinal pigment epithelium
NVU	Neurovascular unit
TJ	Tight junction
AJ	Adherence junctional
DR	Diabetic retinopathy
NPDR	Non-proliferative diabetic retinopathy
PDR	Proliferative diabetic retinopathy
VEGF	Vascular endothelial growth factor
DME	Diabetic macular edema
Ang-2	angiopoietin 2
OCLN	Occludin
CLDN5	Claudin-5
ZO-1	Zonula occludens-1
PKCβ	Protein kinase Cβ
PLVAP	Plasmalemma vesicle-associated protein
IVT	Intravitreal
BBB	Blood–brain barrier
RGC	Retinal ganglion cell
FZD4	Frizzled 4
TSPAN12	Tetraspanin 12
LRP5/6	Low-density lipoprotein receptor-related protein 5/6
APC	Adenomatosis polyposis coli
PP2A	Protein phosphatase 2A
CK1α	Casein kinase 1 α
GSK-3α/β	Glycogen synthase kinase 3α/β
DVL	Disheveled
TCF/LEF	T-cell factor/lymphoid enhancer-binding factor
FEVR	Familial exudative vitreoretinopathy
KO	Knockout
OIR	Oxygen-induced retinopathy
MFSD2	Major facilitator superfamily domain containing 2
ChAT	Choline acetyltransferase
BREC	Bovine retinal endothelial cells
AMD	Age-related macular degeneration
